# Natural history of NF1 c.2970_2972del p.(Met992del): confirmation of a low risk of complications in a longitudinal study

**DOI:** 10.1038/s41431-021-01015-4

**Published:** 2021-12-13

**Authors:** Claire Forde, Emma Burkitt-Wright, Peter D. Turnpenny, Eric Haan, John Ealing, Sahar Mansour, Muriel Holder, Nayana Lahiri, Abhijit Dixit, Annie Procter, Laurence Pacot, Dominique Vidaud, Yline Capri, Marion Gerard, Hélène Dollfus, Elise Schaefer, Chloé Quelin, Sabine Sigaudy, Tiffany Busa, Gabriella Vera, Lena Damaj, Ludwine Messiaen, David A. Stevenson, Peter Davies, Sheila Palmer-Smith, Alison Callaway, Pierre Wolkenstein, Eric Pasmant, Meena Upadhyaya

**Affiliations:** 1 Manchester Centre for Genomic Medicine, Manchester University Hospitals NHS Foundation Trust, Manchester, UK; 2 Clinical Genetics, Royal Devon and Exeter NHS Foundation Trust, Exeter, UK; 3 South Australia Clinical Genetics Services, North Adelaide, SA Australia; 4 Department Of Clinical Genetics, St George’s University NHS Foundation Trust, London, UK; 5 Genetics Service, South East Thames Regional Genetics Service, London, UK; 6 Clinical Genetics Department, Nottingham University Hospitals NHS Trust, Nottingham, UK; 7 All Wales Medical Genetics Service, Cardiff, UK; 8 Service de Génétique et Biologie Moléculaires, Hôpital Cochin, DMU BioPhyGen, Assistance Publique-Hôpitaux de Paris, AP-HP.Centre-Université de Paris, Paris, France and Institut Cochin, Inserm U1016—CNRS UMR8104—Université de Paris, CARPEM, Paris, France; 9 Department of Clinical Genetics, Robert-Debré Hospital, AP-HP and University of Paris-Diderot, Paris, France; 10 Service de Génétique Médicale, CHU Caen, Caen, France; 11 Centre de Référence Pour les Affections Rares en Génétique Ophtalmologique, CARGO, Filière SENSGENE, Hôpitaux Universitaires de Strasbourg; Medical Genetics Laboratory, INSERM U1112, Institute of Medical Genetics of Alsace, Strasbourg Medical School, University of Strasbourg, Strasbourg, France; 12 Service de Génétique Médicale, Hôpitaux Universitaires de Strasbourg, Institut de Génétique Médicale d’Alsace, Strasbourg, France; 13 Service de génétique clinique, CLAD Ouest, CHU Rennes, Hôpital Sud, Rennes, France; 14 Department of Medical Genetics, Children’s Hospital La Timone, Assistance Publique des Hôpitaux de Marseille, Marseille, France; 15 Department of Genetics and Reference Center for Developmental Disorders, Normandy Center for Genomic and Personalized Medicine, Rouen, France; 16 Department of Pediatrics, Competence Center of Inherited Metabolic Disorders, Rennes University Hospital, Rennes, France; 17 Department of Genetics, University of Alabama at Birmingham, Alabama, USA; 18 Division of Medical Genetics, Department of Paediatrics, Stanford University, Stanford, USA; 19 Molecular Genetics, Salisbury NHS Foundation Trust, Salisbury, UK; 20 Département de Dermatologie, AP-HP and UPEC, Hôpital Henri-Mondor, Créteil, France; 21 Division of Cancer and Genetics, Cardiff University, Cardiff, UK

**Keywords:** Clinical genetics, Disease genetics

## Abstract

Individuals with the three base pair deletion NM_000267.3(NF1):c.2970_2972del p.(Met992del) have been recognised to present with a milder neurofibromatosis type 1 (NF1) phenotype characterised by café-au-lait macules (CALs) and intertriginous freckling, as well as a lack of cutaneous, subcutaneous and plexiform neurofibromas and other NF1-associated complications. Examining large cohorts of patients over time with this specific genotype is important to confirm the presentation and associated risks of this variant across the lifespan. Forty-one individuals with the in-frame *NF1* deletion p.Met992del were identified from 31 families. Clinicians completed a standardised clinical questionnaire for each patient and the resulting data were collated and compared to published cohorts. Thirteen patients have been previously reported, and updated clinical information has been obtained for these individuals. Both CALs and intertriginous freckling were present in the majority of individuals (26/41, 63%) and the only confirmed features in 11 (27%). 34/41 (83%) of the cohort met NIH diagnostic criteria. There was a notable absence of all NF1-associated tumour types (neurofibroma and glioma). Neurofibroma were observed in only one individual—a subcutaneous lesion (confirmed histologically). Nineteen individuals were described as having a learning disability (46%). This study confirms that individuals with p.Met992del display a mild tumoural phenotype compared to those with ‘classical’, clinically diagnosed NF1, and this appears to be the case longitudinally through time as well as at presentation. Learning difficulties, however, appear to affect a significant proportion of NF1 subjects with this phenotype. Knowledge of this genotype–phenotype association is fundamental to accurate prognostication for families and caregivers.

## Introduction

Neurofibromatosis type 1 (NF1 [OMIM 162200]) is a multi-systemic, autosomal dominant condition characterised by café-au-lait (CAL) macules, cutaneous neurofibroma (CNF), subcutaneous neurofibroma, plexiform neurofibromas (PNF), axillary and inguinal skinfold freckling, Lisch nodules of the iris and skeletal abnormalities e.g., pseudoarthrosis. Patients with NF1 are at increased risk of developing a variety of tumours including malignant peripheral nerve sheath tumours (MPNSTS), optic pathway gliomas (OPG), astrocytic neoplasms, juvenile myelomonocytic leukaemias (JMML), gastrointestinal stromal tumours (GIST), breast cancers, phaeochromocytomas, duodenal carcinoids, glomus tumours, juvenile xanthogranulomas, and rhabdomyosarcomas. Other associated phenotypes include hyperreflective choroidal spots, short stature, macrocephaly, behavioural, and learning difficulties [1–3].

Traditionally, identification of affected individuals has relied on clinical assessment and diagnosis through standardised NIH criteria [2]. These criteria demonstrate a high positive predictive value for diagnosing adults, who commonly develop cutaneous and subcutaneous neurofibromas, usually from their teenage years [3], but their utility is more limited in the paediatric population, particularly in the absence of a family history.

Although NF1 is a monogenic disorder, it is highly variable, demonstrating inter-familial and intra-familial phenotypic heterogeneity [4]. Moreover, NF1 is a progressive disorder with more features typically developing with age. For many genetic conditions, the correlation of a specific DNA change to disease features is complex. Multiple factors impact phenotype including age-dependent manifestations, allelic and non-allelic heterogeneity, the timing and nature of second hit in specific cells, cellular heterogeneity, wild type allele, epigenetic, reduced penetrance, imprinting, processed and non-processed pseudogenes, regulatory polymorphisms, modifying loci, environment, and stochastic factors. A specific phenotype is determined by the interaction of these factors. Of the more than 3197 different constitutional *NF1* pathogenic variants identified (HGMD), only a very few, including those of codon 1809 and codons 844–848 have established genotype–phenotype correlations. Each of these account for a small proportion of affected patients, totalling approximately 10–15% of people with NF1 [5]. NM_000267.3(NF1): c.2970_2972del p.(Met992del) and missense variants of codon 1809 are associated with a milder phenotype [6, 7] whereas missense variants in *NF1* codons 844–848 and type I microdeletion (1.4 Mb) confer greater risks of severity [5, 8, 9].

The other reported variants associated with genotype-phenotype correlation and not confirmed in large populations include NM_000267.3(NF1):c.3112A>G p.Arg1038Gly [10], missense or splice-site variants in familial spinal neurofibromatosis [11, 12], pathogenic variants (PVs) in 5′ tertile in patients with optic pathway gliomas [13–15], and non-truncating PVs in patients with pulmonary stenosis [16], missense PVs in the cysteine/serine-rich domain (CSRD) [17] and missense PVs affecting p.Met1149, p.Arg1276, and p.Lys1423 [18].

The association of NM_000267.3(NF1):c.2970_2972del p.(Met992del) (hereafter referred to as p.Met992del) with a milder phenotype was first reported in 2007 [19]. Examination of a large number of patients with this specific genotype allows confirmation of previously described phenotypic associations, and features that are consistently absent. A comprehensive study by Koczkowska et al. [20] recently demonstrated a mild phenotype across a cohort of 135 individuals with p.Met992del, with a lack of cutaneous, sub-cutaneous, plexiform and spinal neurofibromas and other malignant complications of NF1 [20].

Here, we present longitudinal data collated from an additional sizeable cohort of 41 individuals (26 familial) from a number of Genetic centres worldwide with p.Met992del.

## Methods

**Fig. 1:**
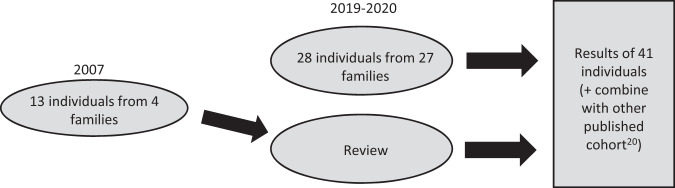
Method of patient ascertainment (molecularly confirmed to have NF1 p.Met992del) for clinical review.

A total of 41 individuals with the same 3-bp deletion p.Met992del were identified following referral to regional genetic laboratories across UK, Europe, USA and Australia. Clinical data were collected between 2018–2020 by referring clinicians using a standardised checklist [7]. Thirteen of these individuals were previously reported by Upadhyaya et al. [19], and for these we present updated clinical information (Fig. 1).

Patients were identified to have a clinical NF1 diagnosis if they met standard NIH criteria [2]. Short stature was defined as a height or length below the 3rd centile, and macrocephaly was defined as a head circumference greater than the 98th centile, comparable to definitions used in other studies [20]. As MRI imaging for optic gliomas and spinal neurofibromas is not routine practice unless a patient displays symptoms or signs, details of any asymptomatic optic gliomas, internal or spinal neurofibromas are less likely to be available. Learning disability was recorded as a manifestation in an individual if detailed by the clinician on the checklist. Intelligence Quotient (IQ) was not assessed formally in the majority of individuals. An individual was considered to have a Noonan phenotype if they had typical dysmorphic features (including hypertelorism, midface hypoplasia, low set ears) or other characteristic manifestations (short stature, a webbed neck or pulmonary stenosis) [7].

Comparisons of our clinical cohort were made with that published by Koczkowska et al. (representing the largest clinical cohort of patients with this phenotype to date) [20] as well as what has been described previously in large molecularly unsegregated NF1 groups [3, 21–25]. Statistics (two-tailed Fisher’s exact test) were performed using GraphPad Quickcalcs [26]. *P* values of <0.05 were considered to be statistically significant.

## Results

### Description of current cohort

Clinical data collected from 41 individuals from 31 families with p.Met992del, including 13 previously described cases in Upadhyaya et al. [19], represent 951.1 person years (defined as the sum of the ages of individuals in the study at the time of review) of follow up for NF1-related complications. The cohort had 21 affected males and 20 females. The majority of individuals were of white ethnic backgrounds (*n* = 25). Twenty-six cases were familial (63%), 11 (27%) sporadic and 4 (10%) unknown. The mean age at clinical assessment was 23.8 years (median age 15.6 years, range 0.3–79.9 years, *n* = 40 [in one case age at assessment was not documented]). Seventeen (41%) individuals were aged 20 years or more at the time of their most recent clinical review and 10 (24%) were over 30 years old. No evidence of mosaicism or a segmental *NF1* phenotype was identified in any individual. Clinical features stratified by age of onset are summarised in Table 1.

**Table 1:** Comparison of frequency of observed features between *NF1* p.Met992del patients in this cohort, the Koczkowska et al cohort [20], and molecularly unsegregated NF1 cohorts detailed in the literature [3, 21–25].

NF1 feature	Frequency						Comparison of current cohort to Koczkowska et al. [20] (*P* value from 2-tailed Fisher’s exact test)	Comparison of those with p.Met992del^i^ with classic phenotype (*P* value from 2-tailed Fisher’s exact test)
	Current cohort				Koczkowska et al. [20] [*n* = 135] (%)	Molecularly unsegregated NF1 patient cohort (%) [3, 21–25]		
	<7 years [*n* = 7] (%)	≥7–18 years [*n* = 16] (%)	≥19 years [*n* = 17] (%)	All* [*n* = 41] (%)				
≥6 Café-au-lait (CAL) macules (HP:0000957)	6/7 (85.7)	14/16 (87.5)	17/17 (100)	38/41 (92.7)	119/135 (88.2)	4058/4419 (91.8) [21, 24]	→*P* = 0.57	→*P* = 0.21
Intertriginous freckling^a^ (HP:0000997 and HP:0030052)	2/7 (28.6)	10/16 (62.5)	13/17 (76.5)	26/41 (63.4)	73/124 (58.9)	3335/4242 (78.6) [21, 24]	→*P* = 0.71	↓*P* < 0.0001
Lisch nodules (HP:0009737)	0/7 (0)	0/11 (0)	2/6 (33.3)^b^	2/25 (8.0)	13/101 (12.9)	2637/3983 (66.2) [21, 24]	→*P* = 0.73	↓*P* < 0.0001
Cutaneous neurofibroma	0/6 (0)	0/16 (0)^c^	0/17 (0)	0/40 (0)	0-1/128 (0-0.8)	336/506 (66.4) [21, 25]	→*P* = 1.00	↓*P* < 0.0001
Subcutaneous dermal neurofibroma (HP:0100698)	0/7 (0)	0/16 (0)	1/17 (5.9)	1/41 (2.4)	0-3/124 (0-2.4)	260/471 (55.2) [21, 25]	→*P* = 1.00	↓*P* < 0.0001
Plexiform neurofibroma (HP:0009732)	0/7 (0)	0/16 (0)	0/17 (0)	0/41 (0)	0/128 (0)	1182/4419 (26.7) [21, 24]	→*P* = 1.00	↓*P* < 0.0001
Spinal neurofibromas^d^ (HP:0009735)	0/0 (0)	0/4 (0)	0/2 (0)	0/6 (0)	0/118 (0)	33/139 (23.7) [21]	→*P* = 1.00	↓*P* < 0.0001
Optic glioma^d^ (HP:0009734)	0/4 (0)	0/8 (0)	0/2 (0)	0/15 (0)	1/164 (0.6)	16/141 (11.3) [21]	→*P* = 1.00	↓*P* < 0.0001
Other neoplasm (HP:0002664)	0/7 (0)	0/16 (0)	1/17 (5.9)^e^	1/41 (2.4)	13/126 (10.3)	397/3643 (19.9) [22]	→*P* = 0.19	→*P* = 0.37
Scoliosis (HP:0002650)	0/7 (0)	4/16 (25.0)	2/17 (11.8)	6/41(14.6)	11/125 (8.8)	101/663 (15.2) [21, 25]	→*P* = 0.37	→*P* = 0.11
Short stature^f^ (HP:0004322)	1/6 (16.7)	1/12 (8.3)	2/9 (22.2)	4/27 (14.8)	11/71 (15.5)	29/124 (23.4) [3]	→*P* = 1.00	→*P* = 0.17
Macrocephaly^g^ (HP:0000256)	1/6 (16.7)	1/7 (14.3)	1/11 (9.1)	3/24 (12.5)	26/87 (29.9)	39/115 (33.9) [3]	→*P* = 0.12	→*P* = 0.25
Pulmonary stenosis (HP:0001642)	0/6 (0)	0/13 (0)	0/16 (0)	0/36 (0)	4/113 (3.5)	25/2322 (1.1) [23]	→*P* = 0.57	→*P* = 0.09
Learning difficulty/cognitive impairment^h^ (HP:0100543)	4/6 (66.7)	10/16 (62.5)	5/17 (29.4)	19/40 (47.5)	50/129 (38.8)	43/138 (31.2) [21]	→*P* = 0.36	→*P* = 0.10

*One age unknown at review

^a^Must be more than one freckle observed to be included.

^b^One patient unilateral, one patient bilateral, total *n* number of patients represents those in which a dedicated eye examination was done.

^c^Neurofibromas had to be histologically confirmed to be included. One patient aged 11 reported to have multiple cutaneous lesions but no histology available.

^d^Frequency only measured from cases who had undergone MRI imaging.

^e^Bladder cancer identified in 70-year-old female.

^f^Short stature defined as height/length <3rd centile, no specific growth charts are available for Asian / Hispanic populations and therefore these individuals were excluded.

^g^Macrocephaly defined as head circumference >98th centile.

^h^One comparator group used for learning difficulty (older classical cohorts not utilised as definitions of learning difficulty have changed over time). One patient excluded as <1 year of age and therefore level of learning difficult to assess.

^i^Koczkowska et al. cohort combined with present cohort.

↑NF1 c.2970_2972del cohort has a statistically significant higher frequency of this feature.

→NF1 c.2970_2972del cohort has an equivalent frequency.

↓NF1 c.2970_2972del cohort has a statistically significant lower frequency of this feature.

**Fig. 2:**
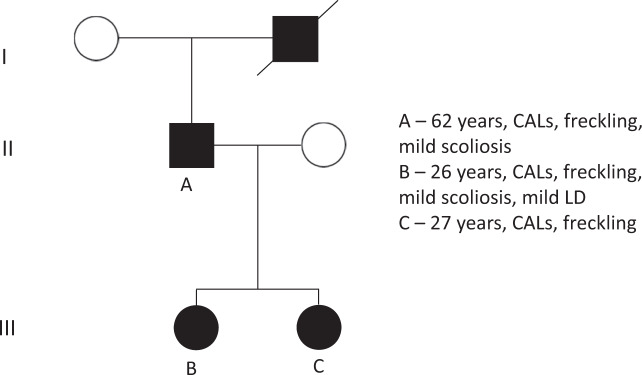
Pedigree of a multigeneration affected family, detailing the phenotype observed.

Of particular interest was the follow-up of 13 patients from four families previously reported [19]. Figure 2 demonstrates a pedigree of one of these multigenerational families and the features observed. The assessment of these individuals has provided insight into long-term outcomes, particularly as these cases comprised 12/17 (71%) of those 20 years old or older in this cohort. All but one of these individuals had an updated clinical evaluation in 2019 representing at least a 12-year interval between initial assessment and follow-up review. It was one of these patients (aged over 50 years) that had the only histologically confirmed intradermal neurofibroma. The eldest member of the cohort, aged 79 years at last clinical review, had a complete absence of any neurofibroma or glioma. This individual developed a bladder cancer at 70 years old.

### Pigmentary features

CALs (38/41, 93%) and intertriginous freckling (26/41, 63%) were commonly observed amongst individuals of all ages in the cohort. Both were present in the majority (26/41, 63%) and the only confirmed feature in 11 (27%) individuals. 34/41 (83%) of the cohort met NIH diagnostic criteria, however this proportion fell to 66% (27/41) when having a first degree relative with an NF1 diagnosis was removed as a criterion.

### Neurofibroma

A 54-year-old female had a single intradermal neurofibroma confirmed by histology. Molecular analysis of this tumour’s DNA was attempted, however, the quality of the DNA extracted was insufficient for next generation sequencing, and therefore the molecular mechanism for its development could not be established.

Multiple ‘cutaneous lesions’ were reported in one other individual (an 11 year old male, for whom longer term follow up data were not available) but these were not histologically confirmed, and therefore a number of aetiologies are possible. No spinal neurofibromas were detected in the five individuals (13.9% of the cohort) who had MRI spinal imaging.

### Ophthalmic features

No optic gliomas were reported, however, only 15 (37%) had an MRI brain scan capable of identifying asymptomatic lesions. Lisch nodules were reported in 2 (of 25) individuals examined (8%). These individuals were from the same family, and in one case they were bilateral. No other ophthalmic features were noted in any patient in this cohort.

### Noonan-like features

Three (7%) individuals were described as having a ‘Noonan-like’ phenotype (*PTPN11* testing was normal in two of these and not performed in the other). One was an 8-year-old male with subtle dysmorphic features—low set ears and a webbed neck (*PTPN11* gene analysis not completed). Another was a 13-year-old male, who had mid-face hypoplasia, short stature (height < 0.4th centile), scoliosis and well defined widespread CALs (14 in total). The final patient was a 40-year-old female, again with short stature and widespread CALs. None of these individuals had a cardiac phenotype such as pulmonary stenosis. A renal pelvis structural abnormality was reported in one individual.

### Other tumours

One patient was diagnosed with bladder cancer at 70 years of age (no information on pathological subtype available), another with a hamartomatous formation near the left thalamus at 7 years old (diagnosed on imaging), and another with a pituitary microadenoma (first identified on scan at 20 years, normal endocrine status). These individual incidences cannot conclusively be associated with p.Met992del at present. Importantly, no patient (despite 17 individuals being included who were over 20 years of age) was diagnosed with a malignant peripheral nerve sheath tumour or other NF1-associated malignancy.

### Neurodevelopmental parameters

Nineteen individuals were described as having a learning disability (19/40, 46%) by the assessing clinician. Within this subgroup, 5 (5/19, 36%) patients were specifically noted to have speech delay. Three individuals in this group also had an official diagnosis of attention deficit disorder. Three people within the cohort as a whole had gone on to achieve a higher degree or had graduated from college.

### Comparison to previously described NF1 p.Met992del cohort

**Fig. 3:**
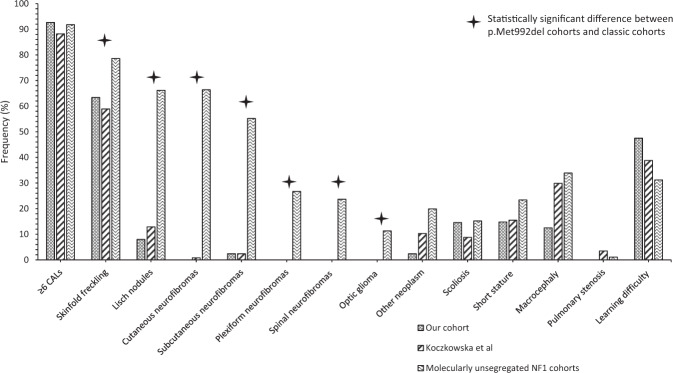
Comparison of frequency of observed features between studied cohort and that from Koczkowska et al. [20], as well as molecularly unsegregated NF1 cohorts detailed in the literature [3, 21–25].

Table 1 and Fig. 3 compare the frequency of observed features in this cohort to that in Koczkowska et al. [20]. No significant differences are evident, with all *P* values >0.05 by two-tailed Fisher’s exact test. This demonstrates a consistency in phenotype between the two cohorts. No formal age-adjusted comparison was possible between cohorts, but the clinical phenotypes in the two studies appear very similar.

### Comparison to classical (clinically diagnosed) NF1 cohorts

Table 1 and Fig. 3 compares the frequencies of the clinical features observed collectively in p.Met992del cohorts to broader groups of NF1 patients, where individuals were not selected by genotype [3, 21–25].

CALs occur at a similar frequency in patients with the *NF1* three base pair deletion as in other NF1 patients (see Table 1, *P* = 0.21), emphasising the significance of CAL in the ascertainment of mild NF1 associated phenotypes. Most other features appear to occur at a lower frequency overall, including neurofibroma of all subtypes and optic glioma. No significant difference was observed between the measured growth parameters of patients (frequency of short stature and macrocephaly appeared similar). Spinal scoliosis, pulmonary stenosis, and learning difficulty appeared to occur at relatively similar frequencies between p.Met992del and the wider NF1 cohort.

## Discussion

We longitudinally studied members of four families (*n* = 13) harbouring p.Met992del [19], as well as an additional 28 new cases. The results of this study accord closely with those of Koczkowska et al. [20] and our earlier findings [19], confirming that individuals with p.Met992del appear to be at a significantly lower risk of external neurofibromas and malignant sequelae of NF1 than other individuals with the condition, even in long term follow-up. Other complications of NF1 for which loss of the wild-type allele has been demonstrated also appear less frequent [27]. In addition, the longterm follow-up findings provide compelling evidence that this mild tumoural phenotype is sustained over a period of at least 12 years. This observed pattern of disease is in contrast with those seen in patients with large *NF1* deletions [8], who frequently exhibit learning disability, and are at higher risk of a range of cardiovascular anomalies, an increased number of cutaneous, subcutaneous, plexiform, spinal and internal neurofibromas as well as an increased lifetime risk of malignant peripheral nerve sheath tumours (MPNSTs) [8].

It is clear that CALs remain a consistent feature in p.Met992del individuals as previously described, and hence represent a firm diagnostic handle from a young age (youngest patient with CALs was 3 months of age). The prevalence of learning disability appears indistinguishable between this genotype and classical NF1, demonstrating the importance of developmental assessment to allow for adequate support. Whilst learning difficulty (LD) appears frequent, the variability of this and the absence of LD in any individual from some larger families studied [19] suggests that additional factors may be important in this context.

It is difficult to clinically differentiate NF1 patients with p.Met992del from Legius syndrome based on skin features as patients with both diseases have overlapping clinical features of CALs, intertriginous frecking and learning difficulty. Revised diagnostic criteria for NF1 and Legius syndrome allow for a more reliable diagnosis of Legius syndrome, in which individuals lack Lisch nodules [28]. Given the phenotypic similarity between these two entities, counselling for affected individuals may be similar.

Four clear NF1 genotype–phenotype correlations, namely microdeletions, p.Met992del, missense variants at Arg1809, and missense variants at codons 844–848, offer biomarkers for clinical management and genetic counselling. Each of these genotypes affects only a small percentage of NF1 individuals, and as such, approximately 10–15% of NF1 patients can be counselled based on their genotype [5]. The future sophisticated methodology combined with the prudent digital revolution will pave the way for identifying additional NF1 genotype–phenotype correlations.

Of the four recognised genotype–phenotype correlations, missense variants affecting Arg1809 cause a NF1 phenotype similar to p.Met992del, demonstrating a lack of neurofibroma and frequency of LD similar to classical cohorts (50%) [6]. An additional small series of patients (*n* = 7) with NM_000267.3(NF1):c.3112A>G p.Arg1038Gly [10], have been reported to have CALs and no neurofibroma, but any comparisons of this small group with p.Met992del must be approached with caution due to small patient numbers.

Several questions remain unanswered from this work. Cutaneous neurofibromas have not been identified in patients with p.Met992del. In the current study, one subcutaneous lesion was detected and confirmed histologically in a patient over 50 years of age. Multiple sporadic neurofibromas have been described in a normal population [29]. Whilst the absence of neurofibromas across the lifespan cannot be assured for any individual, the risk for those with p.Met992del may be similar to the general population baseline (where sporadic neurofibromas occur occasionally with increasing age). It may be that the neurofibroma described had two different inactivating somatic mutations, in addition to the p.Met992del variant identified in the patient’s germline. Notably, there remains a difficulty of clarifying the nature of lesions that are not of specific medical concern and hence may not warrant excision and histological analysis per se.

The molecular mechanism of *NF1* p.Met992del remains unknown [5]. It is intriguing that a 3-base pair (bp) inframe deletion that is predicted to produce a neurofibromin protein with a loss of a single methionine at 992 results in a mild tumoural phenotype, associated with an absence of neurofibroma and optic pathway gliomas. Such observations could probably be explained by a mutant hypomorphic allele (dose effect) that might not confer a sufficient selective advantage to Schwann cells, even with a second inactivating hit of the *NF1* wild-type allele to allow initiation of tumourigenesis. *NF1* somatic mutation has been identified in the melanocytes derived from the café-au-lait macules of unrelated 3-bp deletion patients (Upadhyaya- unpublished work). The hypothesis that the p.Met992del variant may modify specific signalling pathways that would only affect certain phenotypes, whereas the “classical” loss-of-function of *NF1* has effects on additional signalling pathways (and thus a broader effect/phenotype) is currently under investigation by Upadhyaya and collaborators. Similarly, a possible impact of the p.Met992del variant on neurofibromin dimerisation or post-translational modifications (such as ubiquitination or sumoylation) is not yet supported by experimental observations [30]. Additional work is warranted, including functional ex vivo (cell cultures) or in vivo (mouse models) studies.

This is the first longitudinal study confirming the paucity of external neurofibromas and malignancies in NF1 patients with p.Met992del. Long term follow-up (over 900 person years in total) confirms the life-long low risk of debilitating or fatal NF1-associated complications. The phenotype associated with this variant was confirmed in additional patients who became available for evaluation. Although phenotype data was fairly comprehensive, there were limitiations to this work. Caution is warranted in phenotype interpretation as not all features were likely to be systematically assessed unless the patient showed signs or symptoms e.g., structural cardiac disease. As patients were reviewed by a large number of different clinicians, there could be some variability in the assessment of clinical features e.g., Noonan-like dysmorphology, despite best efforts to standardise data collection with a proforma. Learning difficulty with relation to severity can be difficult to assess even with defined parameters e.g., IQ and can vary in presentation across age and sociocultural groups. The high frequency of learning difficulty could result, in part, from ascertainment bias. Individuals with CALs without any additional developmental or health concerns may never present to clinical services. As such, this subgroup may not necessarily be fully represented in our or any clinically ascertained cohort. Finally, comparisons of the described p.Met992del cohort with unsegregated NF1 patient cohorts relied on phenotypic information collated from a limited number of references [3, 21–25] instead of a large unselected registry. Caution therefore must again be applied in interpreting the clinical manifestations that have small denominators (i.e., optic glimoa, spinal neurofibromas, short stature, macrocephaly, and learning problems).

## Conclusion

Individuals with p.Met992del appear to be at a significantly reduced risk of developing visible neurofibromas, malignant sequelae and other significant NF1-related health complications compared to classical, unsegregated NF1 cohorts. Data from our study lend credence to earler findings and further demonstrate that the mild tumoural phenotype associated with *NF1* p.Met992del can persist longitudinally through time. Learning difficulties also appear to be a significant feature in this cohort. Knowledge of this genotype–phenotype association is fundamental to accurate genetic prognostication and counselling for families, and highlights the need for certain interventions e.g., ensuring developmental assessment comprises part of care through childhood.

Although the frequency of this genotypic variant in unrelated, molecularly confirmed, individuals with NF1 is small (0.78% (27/3442) in the NF1 Leiden Open Variation Database (LOVD) database [31] and 0.88%, 74/8400 in University of Alabama cohort [20]), knowledge of this specific genotype–phenotype correlation is important for those families affected as it is possible to provide longterm reassurance about the likely low risk of malignant complications.

## Supplementary information

Data collection proforma

## Data Availability

Phenotypic data of the 28 newly identified patients has been uploaded to the LOVD database (https://databases.lovd.nl/shared/variants/NF1/unique).
