# Supplementary material for: Natural history of NF1 c.2970_2972del p.(Met992del): confirmation of a low risk of complications in a longitudinal study
Source: Eur J Hum Genet. 2021 Dec 13;30(3):291–7. doi: 10.1038/s41431-021-01015-4 (PMC8904810; doi:10.1038/s41431-021-01015-4)
Supplement: Supplementary file 1 — Data collection proforma [file 41431_2021_1015_MOESM1_ESM.pdf]

MEDICAL GENOMICS LABORATORY: *NF1/SPRED1* PHENOTYPIC CHECKLIST FORM

UAB

Patient ID:____

Referring Physician:____ Date of Exam ___/___/___

DEMOGRAPHIC INFORMATION

Gender : ☐ Male ☐ Female Date of Birth: ___/___/___

Ethnicity: Mother: ☐ White ☐Black ☐ Native American ☐ Hispanic ☐ Asian ☐ Other:
Father: ☐ White ☐Black ☐ Native American ☐ Hispanic ☐ Asian ☐ Other:

DIAGNOSIS

NIH criteria:
☐ >6 CAL spots >5mm, postpubertal >15mm ☐ Optic glioma
☐ >2 neurofibromas or 1 plexiform NF ☐ >2 Lisch nodules
☐ Axillary or inguinal freckling ☐ A distinct osseous lesion
☐ First degree relative diagnosed with NF1 by above criteria
Does patient fulfill NIH diagnostic criteria for NF1? ☐ Yes ☐ No

Clinical diagnosis:
☐ NF1 ☐ Multiple CAL spots ☐ Familial multiple CAL spots
☐ Spinal NF ☐ Isolated neurofibromas ☐ Segmental NF1
☐ NF Noonan ☐ Single NF1 feature ☐ Watson Syndrome
☐ Legius syndrome ☐ Unknown

Family history: ☐ Sporadic (proband is a “founder” ☐ Familial (proband is a “non-founder”) ☐ Unknown
Consanguinity: ☐ Yes ☐ No ☐ Unknown

GENERAL INFORMATION

Height: ____cm Head circumference: ____cm Weight: ____kg

NF SIGNS AND SYMPTOMS

1) CAL spots: ☐ 0 ☐ 1-5 ☐ ≥6 to 100 ☐ >100
General impression on the borders of the CAL-spots:
☐ typical well-defined smooth borders diameter:
☐ irregular margins, ragged borders diameter:
for detail on size and location of the CAL-spots and other hyper/hypopigmentation areas: page 3.

2) Skin fold freckling: ☐ None

	Left	Right	Comments (e.g. very faint,…..):
Groin	☐	☐	____________________
Axilla	☐	☐	____________________
Submammary	☐	☐	____________________

3) Lisch nodules: ☐ None ☐ Left ☐ Right ☐ Unknown

4) Cutaneous neurofibromas (soft nodules that project above the skin) : histopathologically confirmed: Y / N

☐ 0 ☐ 2-6 ☐ 6-99 ☐ 100-500 ☐ >500

5) Intradermal neurofibromas (soft depression within the skin w/ pinkish overlying discoloration): histopathologically confirmed: Y / N

☐ 0 ☐ 2-6 ☐ 6-99 ☐ 100-500 ☐ >500

6) Subdermal neurofibromas (firm nodules palpable underneath the skin): histopathologically confirmed: Y / N

☐ 0 ☐ 2-6 ☐ 6-99 ☐ 100-500 ☐ >500

7) Plexiform neurofibromas: histopathologically confirmed: Y / N

☐ None
☐ visible from outside
☐ internal
☐ with hyperpigmentation
☐ without hyperpigmentation

☐ Head ☐ Neck ☐ Trunk ☐ L Arm ☐ L Hand ☐ L Leg ☐ L Foot
☐ Abdomen ☐ Pelvis ☐ Genital area ☐ R Arm ☐ R Hand ☐ R Leg ☐ R Foot

8) Spinal neurofibromas (neurofibromas arising from the spinal nerve root) : histopathologically confirmed: Y / N

☐ Unknown ☐ Absent by MRI ☐ Present, asymptomatic
☐ Present, symptomatic

If present: please provide detail on **figure** page 4
☐ unilateral or ☐ bilateral;
C________ T________, L_________, S_________ regions.

9) Optic glioma: ☐ Unknown
☐ Absent by MRI
☐ Present by MRI, **symptomatic**
☐ Nerve (L and/or R)
☐ Chiasm
☐ Present by MRI, **asymptomatic**
☐ Nerve (L and/or R)
☐ Chiasm

10) Other neoplasms: ☐ None
☐ Hypothalamic glioma ☐ Brainstem glioma ☐ Other glioma
☐ MPNST ☐ JMML ☐ Rhabdomyosarcoma
☐ Pheochromocytoma ☐ Colonic polyps ☐ Lipoma
☐ juvenile xanthogranuloma ☐ Breast cancer
☐ schwannoma ☐ Other, specify:______________________________

11) Skeletal Abnormalities: ☐ None
☐ Long bone dysplasia ☐ Pseudoarthrosis ☐ Sphenoid wing dysplasia
☐ Bone cysts ☐ scoliosis ☐ Dysplastic vertebrae
☐ pectus excavatum ☐ pectus carinatum ☐ Other:________________

12) Cardiovascular disease: ☐ Absent
☐ Unknown
☐ Present: ☐ Hypertension ☐ Pulmonic stenosis ☐ Aortic stenosis
☐ Renal artery stenosis ☐ Moya moya ☐ ASD
☐ VSD ☐ Other_________________

13) Development: ☐ Normal ☐ Exam not done

☐Abnormal ☐ ADD ☐ Hyperactivity ☐ Learning disability
☐ speech delay ☐ autism ☐ pervasive developmental delay
IQ: Full scale _______, Verbal ________, Performance ________.

14) Education: ☐ Too young for school ☐ At or above age level ☐ Below age level ☐ Unknown
☐ HS completion ☐ College graduate ☐ Higher degree

15) Noonan phenotype:

☐ Absent ☐ Possible ☐ Unknown

☐ Present:
- ☐ Short stature ☐ Low set ears
- ☐ Hypertelorism ☐ Webbed neck
- ☐ Midface hypoplasia
- ☐ PTPN11 testing done? Result ___________
- ☐ Pulmonic Stenosis
  - peak jet velocity ____m/s
  - peak gradient _____mmHg across pulmonic valve

16) Segmental NF phenotype: ☐ Absent ☐ Possible

location/size of pigmentary lesions and/or neurofibromas

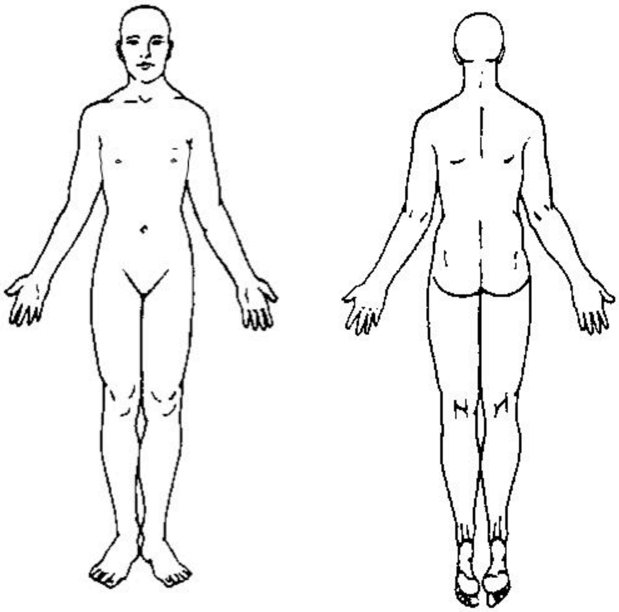

Indicate size and location of

Neurofibromas

CAL-spots

Freckling

Hyperpigmented region

17) location of spinal tumors (if present)

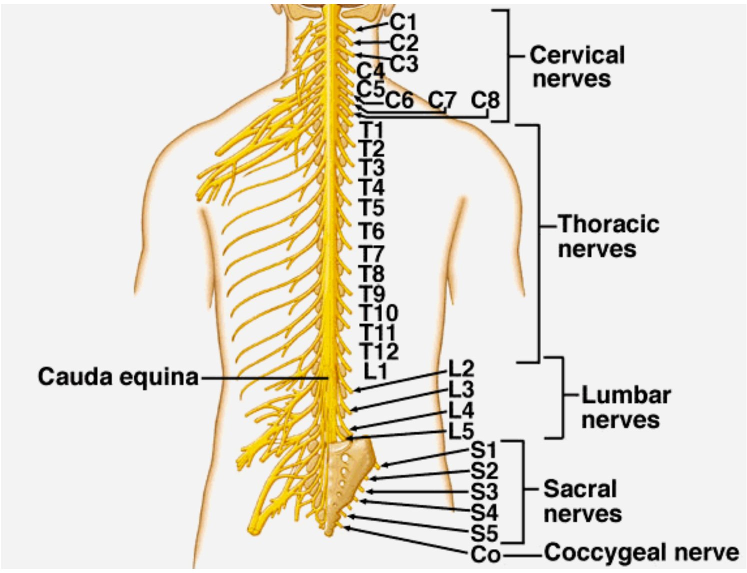

18) any other comments/remarks:
